# If You’re House Is Still Available, Send Me an Email: Personality Influences Reactions to Written Errors in Email Messages

**DOI:** 10.1371/journal.pone.0149885

**Published:** 2016-03-09

**Authors:** Julie E. Boland, Robin Queen

**Affiliations:** 1 Department of Psychology, University of Michigan, Ann Arbor, Michigan, United States of America; 2 Department of Linguistics, University of Michigan, Ann Arbor, Michigan, United States of America; Kyoto University, JAPAN

## Abstract

The increasing prevalence of social media means that we often encounter written language characterized by both stylistic variation and outright errors. How does the personality of the reader modulate reactions to non-standard text? Experimental participants read ‘email responses’ to an ad for a housemate that either contained no errors or had been altered to include either typos (e.g., *teh*) or homophonous grammar errors (grammos, e.g., *to/too*, *it’s/its*). Participants completed a 10-item evaluation scale for each message, which measured their impressions of the writer. In addition participants completed a Big Five personality assessment and answered demographic and language attitude questions. Both typos and grammos had a negative impact on the evaluation scale. This negative impact was **not** modulated by age, education, electronic communication frequency, or pleasure reading time. In contrast, personality traits **did** modulate assessments, and did so in distinct ways for grammos and typos.

## Introduction

Social media may be characterized as including styles of writing that differ from standard usage conventions [1–3]. For instance, variation in punctuation and spelling conventions (using single graphemes like *u* instead of multiple graphemes like *you*) is becoming increasingly visible in different channels of electronically mediated communication. This variation is not necessarily evidence of an error, even though people may express a variety of opinions about it. This variation exists in the same general spaces as actual errors and judgmental comments about both types of deviations from standard usage are also common [4]. The pair of comments below, found on Jezebel.com in response to a parenting article represent a case of the latter:

A: *Homeschooling should be illegal*

B: *Often by parents who don't even know the difference between "there" and "their"*.

Despite the frequency of such comments, linguists who have addressed these kinds of sentiments often attribute them to “peevers” or “grammar police,” and consider them to be primarily motivated by inaccurate assessments of what constitutes a linguistic error (e.g., [5]). In other words, there is an assumption that there is little of specifically linguistic interest involved. Few studies concerning the interpretation of such errors has to date been undertaken (see however [6]). In this paper, we focus specifically on actual written errors (not changing conventions of writing) and the ways in which social interpretations of them may be influenced by characteristics of the listener.

While formal written language changes much more slowly than spoken language (e.g., allowing us to read Shakespeare with little difficulty), social media has brought increased variability in writing. In social media venues, stylistic variation may function much like spoken language variation—to establish personal and group identity [**7**]. For example, in both their spoken language and in their text messages, teenagers may adopt linguistic conventions that are shared among their peers, but differ from those of their parents [**7**].

Social assessments of written errors take on particular importance given that many of our interactions either occur solely via electronically mediated communication (EMC) or become face to face interactions only after initial vetting via EMC. This is true of interactions with people we know and, of particular interest in this study, with people we don’t know. Examples range from getting a loan from Lending Club to starting a romantic relationship via online dating. When we interact electronically with people we don’t otherwise know, the effects of written errors may be heightened because of the lack of the kinds of contextual information found in face-to-face interaction. In an experiment that simulated vetting a potential new colleague, unknown strangers were perceived to be less conscientious, intelligent, and trustworthy when the person was represented by a single email message containing many grammatical errors (such as incorrect use of homophones and subject-verb agreement errors), compared with a content-matched email message without the errors [8]. Likewise, marketing scholars have found that both assessments of believability and actual consumer purchasing are affected by whether or not written copy (ad copy or reviews) contains errors [9–10].

In this study, we have two primary interests in written errors. First, do people react to different types of errors differently and second, are there individual differences that affect the impact of written errors? In considering individual differences, we examined personality traits and patterns of engagement with formal vs. informal written language.

Our interest in personality is motivated, in part, by the literature indicating the important role of personality traits in the production of language (e.g., [11–12]). For example, Park and colleagues used a large sample of Facebook users who had completed a Big Five personality survey to develop a mathematical model that successfully predicted personality traits in other Facebook users, based solely on the language used in their Facebook status updates [12]. Similarly, James Pennebaker and colleagues have shown a variety of correlations between personality traits and the use of words having a more or less positive valence [13–15]. Yarkoni claims, for instance, that “personality plays a relatively pervasive role in shaping the language people use, and that diffuse associations with both function and content words can be reliably identified given a sufficiently large dataset” ([16], p. 371).

If personality influences what people produce, personality might also influence how text is interpreted. We are not suggesting that personality shapes the basic cognitive mechanisms that use grammatical knowledge to produce and understand sentences. Rather, personality may shape certain pragmatic and/or habitual aspects of language use, such as a tendency to write about positive life events, or a tendency to react negatively to certain usage patterns. Because language production and comprehension rely on at least some shared representations and mechanisms [17], we would expect to see reflections of personality in both, rather than solely in production. Indeed, a few studies have shown that individual differences in empathy influence some aspects of language interpretation [18–19]. However, we know of no research investigating how the Big Five personality traits shape any aspect of language comprehension. We focused specifically on one aspect of interpretation, by investigating the reactions elicited by written errors. We chose this specific domain for two reasons. First, peever reactions to non-standard grammar are often ascribed especially to curmudgeonly personalities, and thus we expected reactions to vary with personality traits. Second, typos and grammatical errors—while similar in many ways—may have different social meanings, and therefore should interact with personality traits in distinct ways.

In this paper, we ask whether people’s assessments of writers are affected by written errors when the message is held constant. We compare mechanical errors linked to keyboarding (“typos,” for instance *abuot* for *about*) and traditional peever errors that are only relevant in written language (“grammos,” for instance selecting the wrong form of *to/two/too*). The context in our experiment is a short email-type response to a housemate ad.

We hypothesized that there are different attributions associated with grammos and typos. Typos are often attributed to carelessness and clumsy or hurried typing, rather than ignorance of spelling conventions. Consider the common typo *teh* for *the*. When we encounter this typo, it doesn’t occur to us that the writer doesn’t know how to spell *the*; instead we assume that error was caused by a mechanical problem, maybe the writer was typing in a rush or using a sub-optimal keyboard. In contrast, when we encounter a grammo, like *to* for *too*, we may wonder if the writer is ignorant of the *to/too* distinction. If so, the attributions associated with grammos are more personalized and may thus be more likely to impact other unrelated assessments of the writer (such as trustworthiness), compared with the more neutral attributions associated with typos.

Kreiner and colleagues provided some evidence that typos have more neutral attributions than other types of spelling errors [20]. They found that typos like *cetnered* affected ratings of writing ability less than phonological errors like *sentered* when there were 12 such errors in a 210 word essay. However, the same contrast was not observed when there were fewer errors [20]. In our own previous research, we found that both typos and grammos (2 to 4 errors in 50 to 90 word messages) impacted readers’ assessments, but that grammos impacted both social and academic assessments, whereas typos primarily impacted academic assessments [6].

In the current paper, we explore the degree to which characteristics of the readers themselves modulate the negative impact of errors on assessments of the writer. As an expansion to our earlier study in which we asked participants about various attitudes and behaviors [6], in this study we measured personality traits of our participants as well. We predicted that personality traits would produce both main effects and interactions with errors. For example, we expected more positive ratings overall from participants who are more agreeable, whereas we expected highly conscientious participants to show a larger decrease in evaluations to error-ridden paragraphs compared with error-free paragraphs. Furthermore, if typos and grammos have different social meanings, we would expect to see different patterns of personality interactions for the two types of errors.

## Method

### Participants

Eighty-three native English speakers were recruited via Amazon’s Mechanical Turk (MTurk) and completed the experiment for a fee of $1 each. All participants worked from IP addresses in the US. Our target of 80 participants was somewhat arbitrary because we lacked estimates of effect sizes for the personality variables. Our previous research used 30 college students per experiment, but did not measure personality traits [6]. For this study, we opted to more than double the sample size, given the expected greater variability of MTurk participants.

### Language Stimuli

The primary task in this experiment involved reading simulated email responses to an ad for a housemate, and evaluating the writer on both social and academic criteria. The email stimuli were manipulated such that the same content was presented in one of three written conditions: without errors, with grammatical errors only, or with typos only. The words that created the grammatical errors were always homophonous with the correct word (e.g., replacing *you’re* with *your*).

Both grammos and typos can be considered spelling errors, and in both cases the actual output is orthographically similar to the intended output. However, there are important differences. Grammos violate syntactic constraints, producing a sentence that is ungrammatical in its written form, whereas typos violate lexicality constraints, producing a non-word. Furthermore, the grammos in this study were always pronounceable whereas many of the typos were not.

In designing our stimuli, we tried to create typos and grammos that were similar to errors we have actually encountered. However, it is not possible to equate the frequency of the letter-strings comprising the typos and grammos because of the lexicality difference: words will always be more frequent than non-words. Because the letter strings in the typos were less frequent and more likely to violate pronunciation constraints than the grammos, typos may be more noticeable to readers. In previous research, we tested this hypothesis with an editing task and found that indeed, our typos were more noticeable than our grammos [6].

The twelve paragraphs used for the primary task were taken from prior research [6]. An example is given below. The full set of items is available in the online Supporting Information, S1 Appendix. Each paragraph had three versions, corresponding to the three experimental conditions: fully correct, 2–4 grammos (underlined), or 2–4 typos (in boldface). As described in the Procedure section, each participant read only one version of a given paragraph. The simulated respondent’s name was included in each paragraph; in an effort to avoid gender stereotyping, each name was unisex.

**Hey! My name is Pat and I’m interested in sharing a house with other students who are serious** abuot (about) **there (their)** **schoolwork but who also know how to relax and have fun. I like to play tennis and love old school rap. If** **your (you’re)** **someone who likes that kind of thing too, maybe we would** mkae (make) **good housemates.**

### Scales

Three questionnaires were used in this study. First, a demographic/behavior questionnaire asked about age, gender, first language, highest education level, number of texts per day (0 to 100), features used on Facebook (chat, private message, wall posts, other) and frequency of usage, time spent pleasure reading, and the importance of good grammar. For education level, participants selected among (1) High School Diploma or below, (2) Some College, (3) Associate’s Degree, (4) Bachelor’s Degree, (5) Master’s or Professional Degree, and (6) Ph.D. The Facebook items were each queried on a seven-point scale, from never to daily.

Second, participants completed a 44-item version if the Big Five Personality index (BFI) [21–22]. This index includes scales for extraversion, agreeability, conscientiousness, neuroticism, and openness.

Third, after reading each paragraph (described above, under Language Stimuli), participants completed the same short questionnaire (i.e., the Housemate Scale). A 7-point Likert-type scale was used, with 1 labeled *strongly disagree* and 7 labeled *strongly agree*. Intermediate numbers were not labeled. The items in the assessment questionnaire are listed in Table 1. This scale is a slightly modified version of the scale developed by Queen and Boland [6]. For the current study, we used the ten items that provided assessment of the writer, omitting the single item that focused on the writing itself.

**Table 1:** Questionnaire Items for each Paragraph.

Question	Comments
I think I would be friends with this person	
The writer would be a good housemate.	
The writer seems a lot like me.	
The writer seems friendly.	
The writer seems more sophisticated than most of my friends.	
The writer seems less intelligent than most of my friends.	Reverse-coded
The writer seems conscientious.	
The writer seems considerate.	
The writer seems likeable.	
The writer seems trustworthy.	
This email flowed smoothly.	Not used in Housemate Scale

## Procedure

Both informed consent and the data collection were handled via Qualtrics. On the first screen, participants read the following text and checked a box (yes or no), giving consent to complete the experiment.

Thank you for participating in this study. We will begin by asking you some questions about yourself. We are collecting demographic information (age, gender, etc), information about your habits (e.g., how much do you read? how much do you text?), and information about what you are like as a person (e.g., are you talkative? are you careless?). It is important that you answer all of these questions honestly and reflectively. Some people may not feel comfortable providing this kind of personal information. If you don't feel comfortable answering these types of questions, you won't be able to complete the study. You can opt out now, or at any point during the study. However, if you opt out, we won't be able to pay you. In order to receive payment, we ask that you answer every question. During the second part of the study, we ask you to imagine that you are a college student, looking for a housemate. You will be reviewing 12 "emails" from housemate candidates. After reading each "email", we will ask you to evaluate the writer on a set of dimensions (e.g., do they seem friendly? do they seem conscientious?).

The experiment had four parts in the following order, a demographic/behavior questionnaire, a personality assessment, the primary task (reading and evaluating 12 emails), and wrap up questions. The University of Michigan Institutional Review Board reviewed this experiment, including the consent procedure and determined that the study was exempt from review (the official waiver is provided in the Supporting Information as the S2 Appendix). However, except for the personality test, all components of the experiment had been approved by the University of Michigan Institutional Review Board for in-person experiments [6]. Both the questionnaire and the personality test provided individual difference variables to predict how the emails would be evaluated on the Housemate Scale.

For the primary task, participants were told that the paragraphs were email responses to an ad for a housemate. Participants were randomly assigned to one of three participant groups. Each group read the same 12 paragraphs in random order, but the conditions of the paragraphs were rotated across groups in a Latin square design. Each participant read four paragraphs in each condition and each paragraph was read by roughly equal numbers of participants in each condition. Following each paragraph, participants completed a short questionnaire (i.e., the Housemate Scale) about the writer.

Finally, at the end of the experiment, we asked participants “Did you notice any grammatical errors in the responses you read? (yes/no)” and if they responded affirmatively, we asked, “How much did the errors bother you? (none, little, some, a lot).” Participants completed the demographic/behavior questionnaire, the BFI, the paragraph assessments and the wrap up question(s), all in 10 to 20 minutes.

## Results

All data from all participants were included in the analysis. The only missing data are for the two wrap-up questions, which participants were not required to answer. All other experimental prompts required a response.

### Demographic & Behavior Questionnaire

The results of the participant questionnaire, as well as the final two questions about noticing and being bothered by errors in the paragraphs are summarized in Table 2. Questions about texting and Facebook usage were collapsed into a single EMC scale, based on the following rationale. After converting the number of texts per day to a seven point scale, the Chronbach's alpha for texting and the four Facebook items was .79, indicating that these responses were highly intercorrelated. Exploratory factor analysis revealed a single underlying factor.

**Table 2:** Demographic, Attitude, and Behavioral Data about the Participants.

Question	Mean	% or Range	Comment
Gender		64% women	
First Language		96% American English, 4% Other English	
Highest Education Level		47% Bach., 24% some college, about 10% each in high school, Assoc., and Master's/Prof.	
Age	32.7	19–62	
Pleasure Reading	5.0	1–7	1 = never
EMC	3.52	1–6.5	1 = low
Grammar Attitude	5.88	3–7	1 = not at all important
Notice Errors?		81% yes	
Bothered by errors?	2.85	1–4	1 = not at all

As expected, participants who reported grammar being more important at the beginning of the experiment were somewhat more likely to report being bothered by grammatical errors at the end, although the correlation was low (R = .33).

### Personality Test

Mean scores on the five subscales of the BFI are presented in Table 3, with Cronbach’s alpha as a measure of the internal reliability on each subscale. The index uses a 5-point scale, with 5 representing a high degree of the personality feature and 1 representing a low degree of the personality feature. Correlations between demographic variables and BFI scores are available in the S1 Table of the online Supporting Information. Although we expected some of the demographic measures would be correlated with personality traits, we observed only modest correlations.

**Table 3:** Mean Score and Chronbach's Alpha for each Big Five Trait.

Trait	Mean	Standard Deviation	Alpha
**Extraversion**	3.05	.77	.86
**Agreeable**	3.69	.62	.84
**Conscientious**	3.92	.71	.90
**Neurotic**	2.48	.87	.92
**Open**	3.68	.64	.87

### Participant Assessment of Emails

The first 10 items from Table 1 were averaged into a single scale, called the Housemate Scale, with higher ratings reflecting greater overall positivity toward the message writer (range was 1 to 7). These 10 items all refer to characteristics of the writer, whereas the final item refers to processing ease. We considered using the final item as an independent estimate of processing difficulty, but ended up discarding it out of concern that it was too strongly influenced by the responses to the other items. While we originally selected the items on the scale to measure different aspects of paragraph assessment (e.g., grammos might make a writer seem less intelligent but more friendly), in practice, the items were much too highly intercorrelated for such distinctions, with a Chronbach’s Alpha of .92. Summary statistics are provided in the S2 Table of the online Supporting Information. An exploratory factor analysis with orthogonal rotation revealed a single latent factor (using the criterion that Eigenvalues be above one) that explained 53% of the variance. Removing the three items with loadings below .4 (intelligent, sophisticated, and friendly) increased the explained variance of that single factor to 64%. (Chronbach’s alpha for the 6-item set remained at .92.) Thus, while our 10-item Housemate scale is not strictly unidimensional, it is probably best understood as a coarse-grained assessment of the overall positive/negative attitude of the reader toward the writer, immediately after reading the paragraph.

A set of linear mixed effects models were fitted to the data, using the lmer function in the lme4 package of R version 3.2.1. The Housemate Scale score (for each trial) was the dependent variable. The goal of these analyses was to understand how the linguistic variables (typos and grammos) and the participant variables (e.g., demographics, personality) influenced evaluations of the paragraphs. Participant and item were included as random effects, with random intercepts. All participant variables were centered prior to analysis. Confidence intervals (95%) were generated using the lme4 confint function, “wald” method. Statistical significance was evaluated by using the anova function to generate F statistics. F values were considered significant if they were above 4.00. Because there is no clear means of determining the degrees of freedom within a linear mixed effects model, we took a conservative, but necessarily arbitrary, approach to specifying the degrees of freedom (1,60) and set alpha to .05. Effects labeled as marginal had F values above 2.79, reflecting an alpha of .10.

For Model 1, no personality variables were included. Like prior reported models [6], it assesses the impact of grammos and typos without considering the role of personality. This was important because the current study includes participant variables that were not included in prior models [6], and we wanted to replicate the general findings reported in Queen and Boland [6]. Fixed effects were the number of Typos in the paragraph (0 to 4), the number of Grammos in the paragraph (0 to 4), and five participant variables: EMC score, Age, Education Level, Grammar Attitude and time spent Pleasure Reading. The results are summarized in the S3 Table of the online Supporting Information. There were main effects of both Typos and Grammos. None of the included participant variables affected paragraph ratings overall (e.g., older participants were not any more negative in their assessments than younger participants), but the participant’s Grammar Attitude interacted with Typos and the participant’s Education Level marginally interacted with Grammos. The significant effects are described below, in the context of the best-fitting model, which included the personality variables.

We created two additional statistical models, using the personality variables. The simple personality model included the personality variables crossed with the number of Typos and the number of Grammos, without any of the other participant variables that were used in the model described above. The complex personality model included the five personality variables crossed with Typos X Grammar Attitude and Grammos X Education, i.e. the two interactions observed in the original model. The complex model fit the data better than either Model 1 or the simple personality model (using the anova function to compare model fits), so it is described in Table 4. The amount of variance explained by the final model was also evaluated using the r.squaredGLMM function from the MuMIn package. The conditional r^2^ (combining both fixed and random effects) was .50 and the marginal r^2^ (fixed effects only) was .13.

**Table 4:** Summary of the Primary Statistical Model, including Personality Variables.

Effect	Estimate	95% Confidence Intervals		F
**Typos**	**-.16**	**-.20**	**-.10**	**19.87**
**Grammos**	**-.07**	**-.11**	**-.04**	**16.50**
**Typos X Grammar Att**	**.08**	**.03**	**.14**	**5.34**
Grammos X Education	-.02	-.05	.00	1.79
**Agreeable**	**.30**	**.07**	**.53**	**14.58**
Conscientious	.15	-.07	.37	0.01
Extraversion	.05	-.25	.14	1.41
Neurotic	.08	-.10	.27	0.77
Openness	.06	-.17	.28	1.55
**Grammos X Agreeable**	**.05**	**-.01**	**.12**	**4.18**
**Grammos X Extraversion**	**.03**	**-.03**	**.08**	**4.53**
**Typos X Extraversion**	**.07**	**.01**	**.13**	**6.12**
**Typos X Conscientious**	**-.08**	**-.16**	**.00**	**5.41**
**Typos X Openness**	**.04**	**-.04**	**.12**	**4.59**
**Typos X GramAtt X Extraver**	**.06**	**.01**	**.12**	**8.05**

*Note:* Significant effects and interactions are bolded. Non-significant interactions with personality traits are omitted.

**Fig 1:**
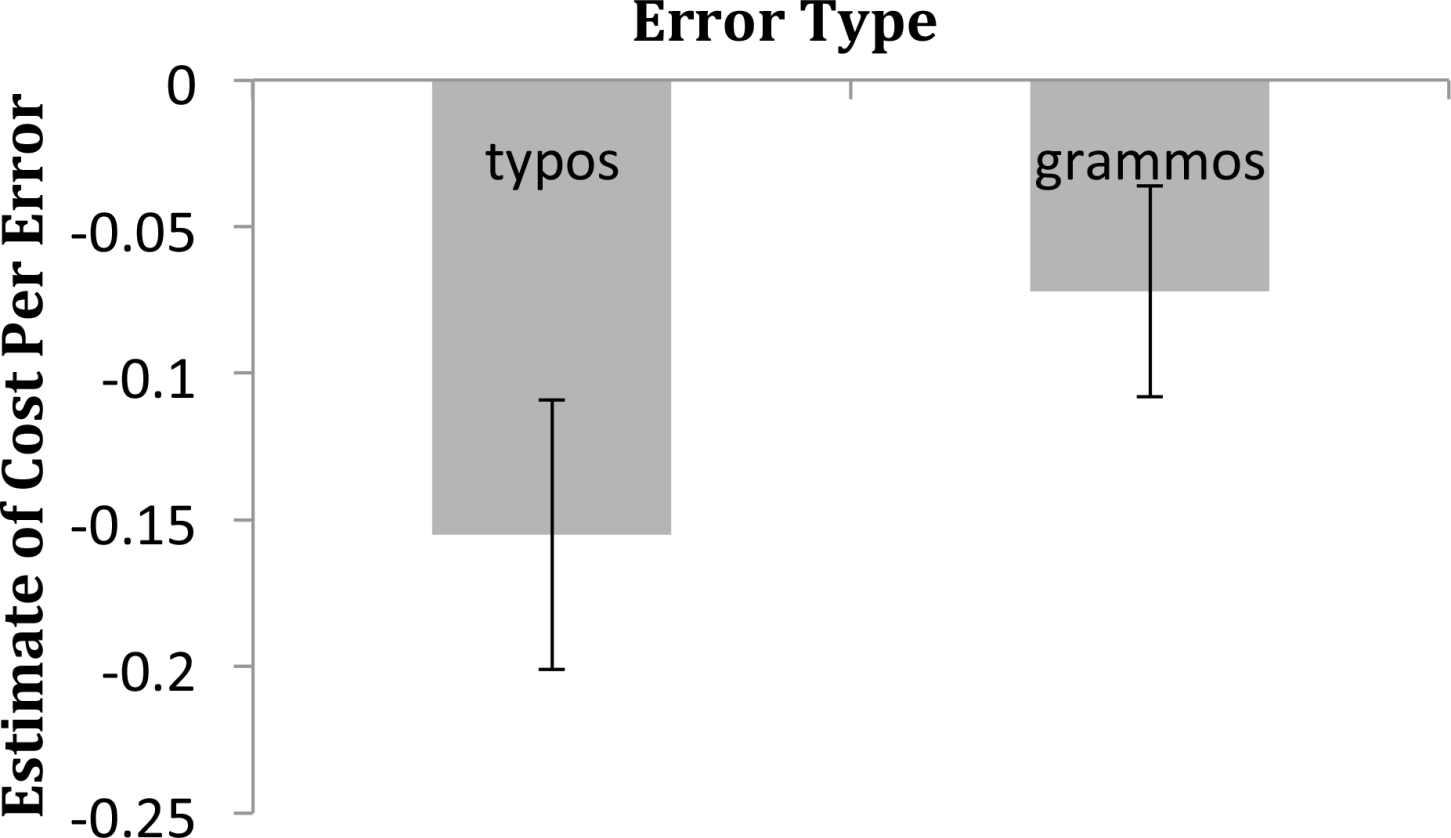
The effects of Typos and Grammos on the Housemate Scale. Point estimates and 95% confidence intervals are taken from the model summarized in Table 4. Because traditional measures of effect size are not suitable to the statistical model used here, parameter estimates are used to indicate effects and confidence intervals provide an estimate of precision (see [23]).

The significant effects from Model 1 were all observed in the full model. Fig 1 illustrates the cost of Typos and Grammos on the Housemate Scale. Typically, error trials contained three errors, so a typical Typo trial was rated .47 lower than a fully correct trial on the 7 point scale, and a typical Grammo trial was rated .22 lower. The cost of a Typo interacted with Grammar Attitude, such that participants who indicated that grammar was most important to them (a 7 on the 7-point scale) reacted less strongly to typos than participants who indicated that grammar was less important. The marginal Grammos X Education interaction from Model 1 did not approach significance in the full model.

**Fig 2:**
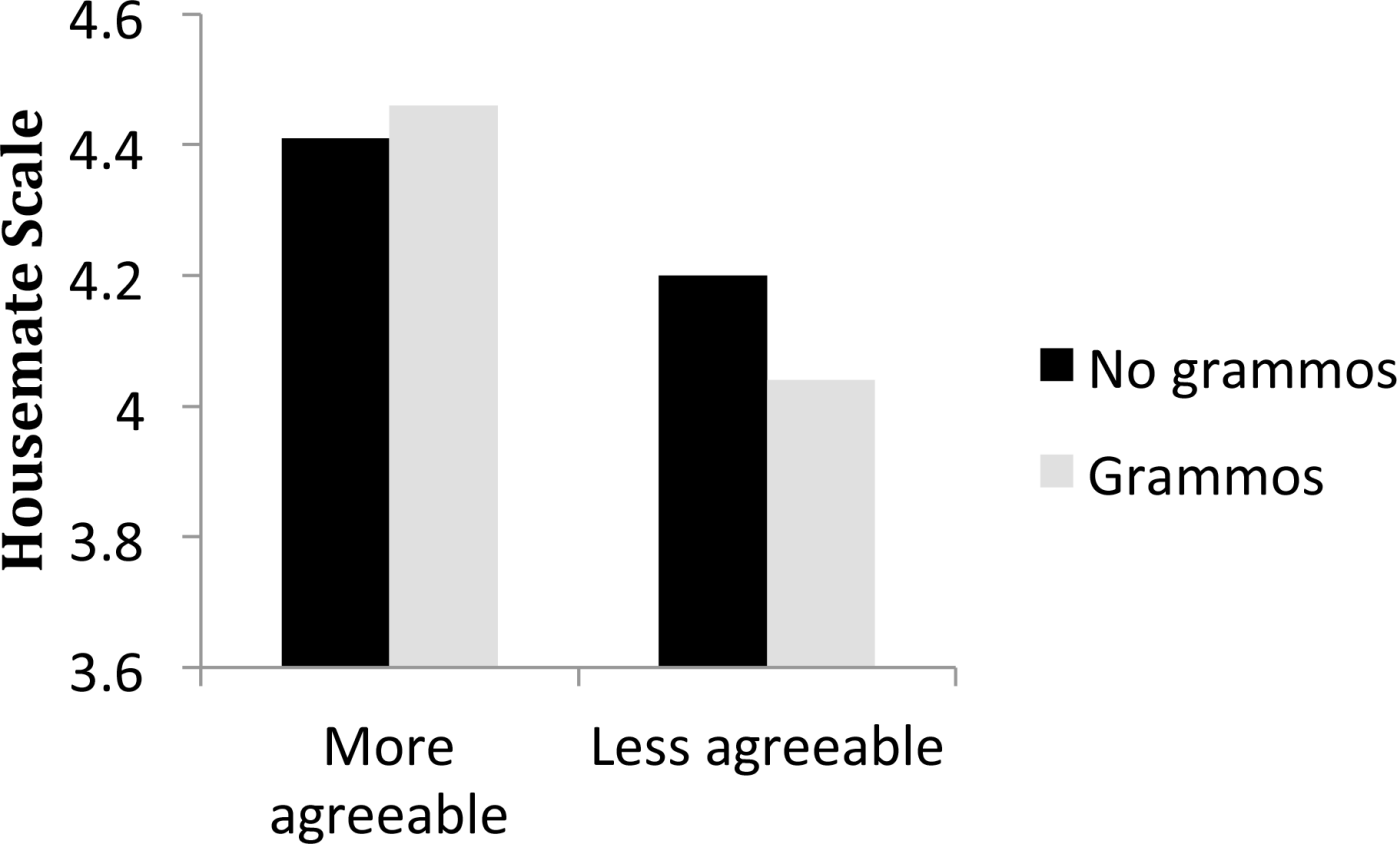
The effect of Agreeability on the Housemate scale, and the interaction between Grammos and Agreeability. Participants were split into two groups at the median of Agreeability (3.78). The more agreeable group averaged a score of 4.21 on the agreeable scale of the BFI, while the less agreeable group averaged 3.21.

**Fig 3:**
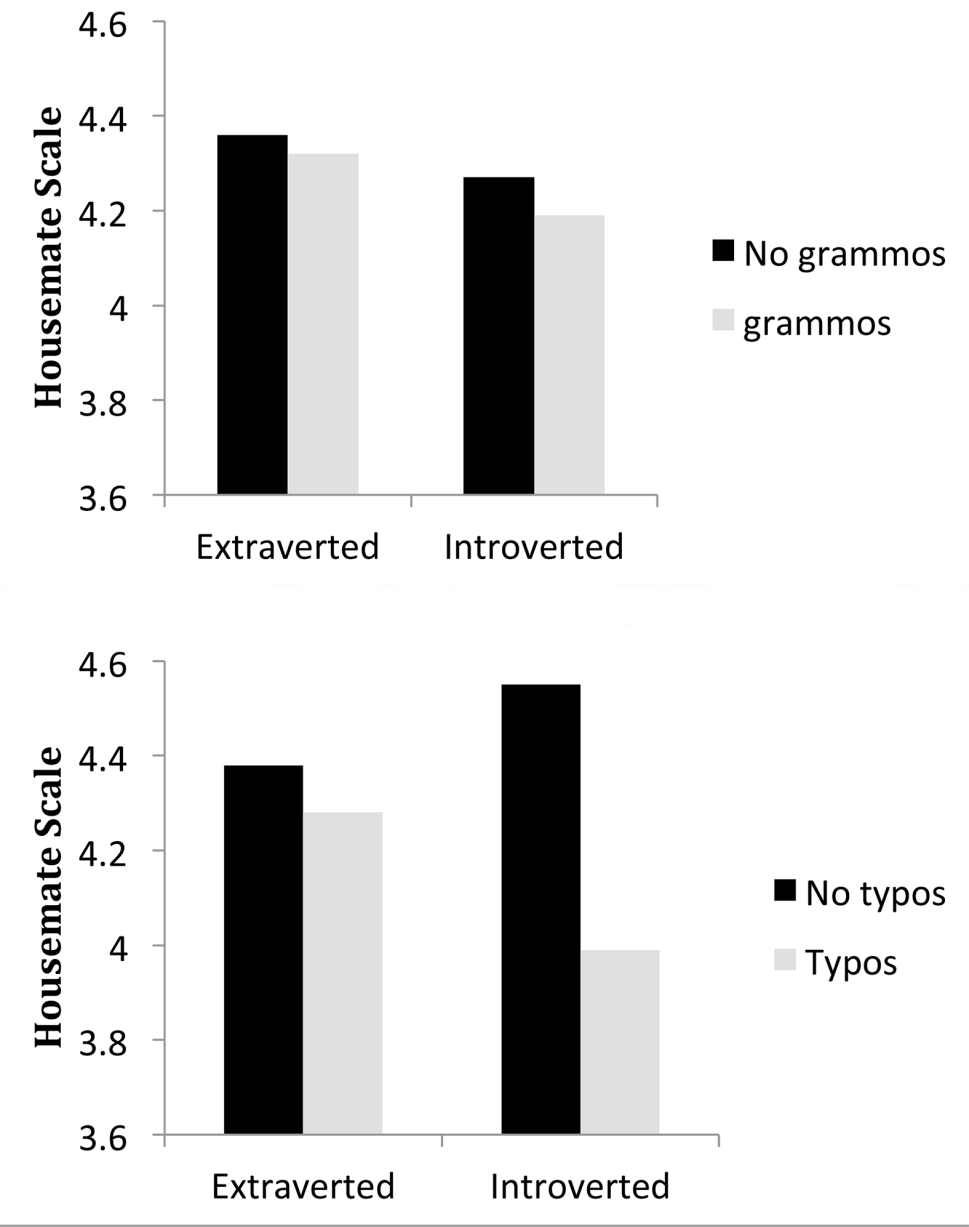
The effect of the Extraversion trait on grammos (top) and typos (bottom). Participants were divided into two groups at the Extraversion median of 3.125. The extraverted group averaged 3.80 on the Extraversion index of the BFI and the introverted group averaged 2.45.

**Fig 4:**
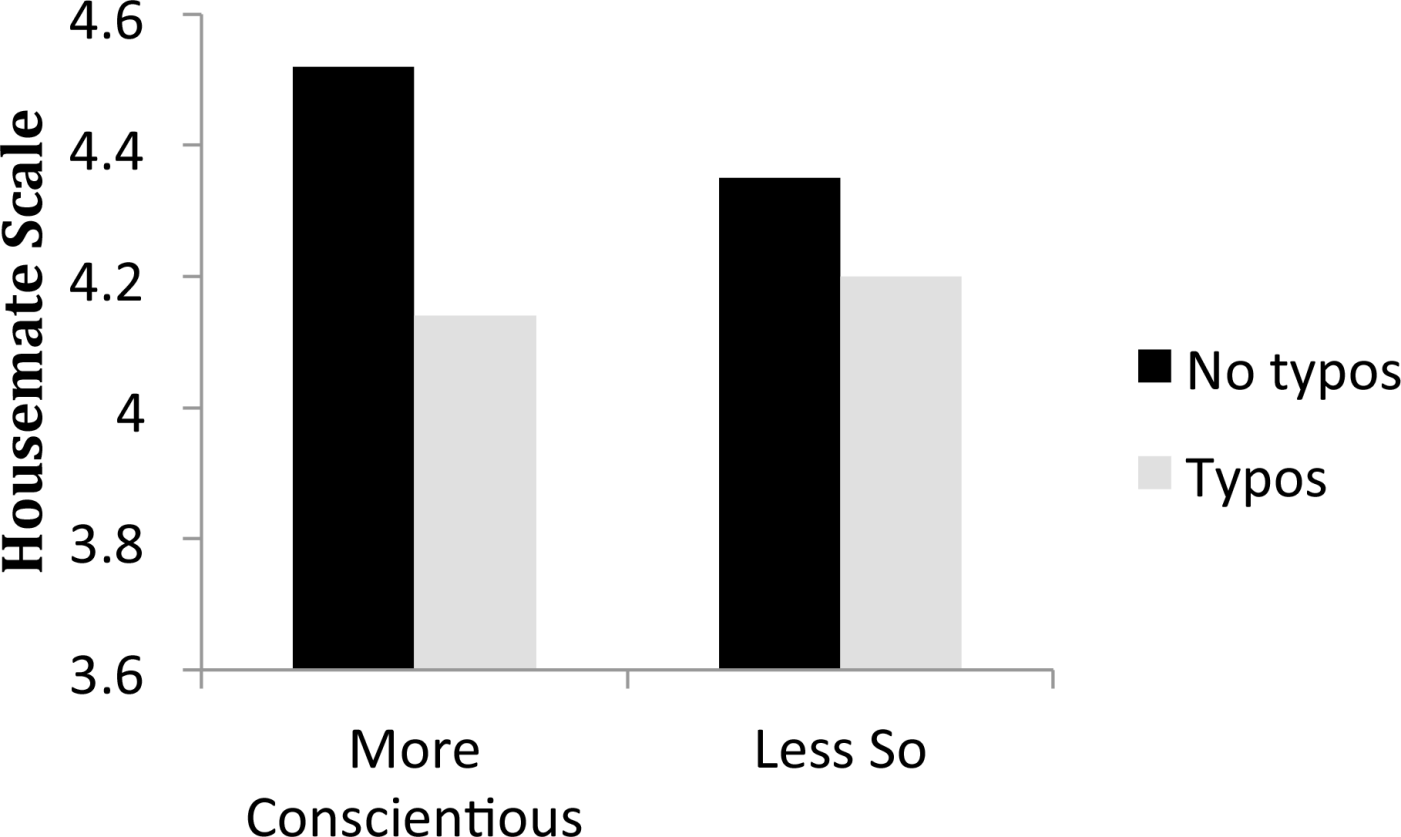
The interaction between Typos and Conscientiousness. Participants were divided into two groups, using the Conscientiousness median of 4.00. The more conscientious group averaged 4.61 on the Conscientious subscale of the BFI and the less conscientious group averaged 3.30.

**Fig 5:**
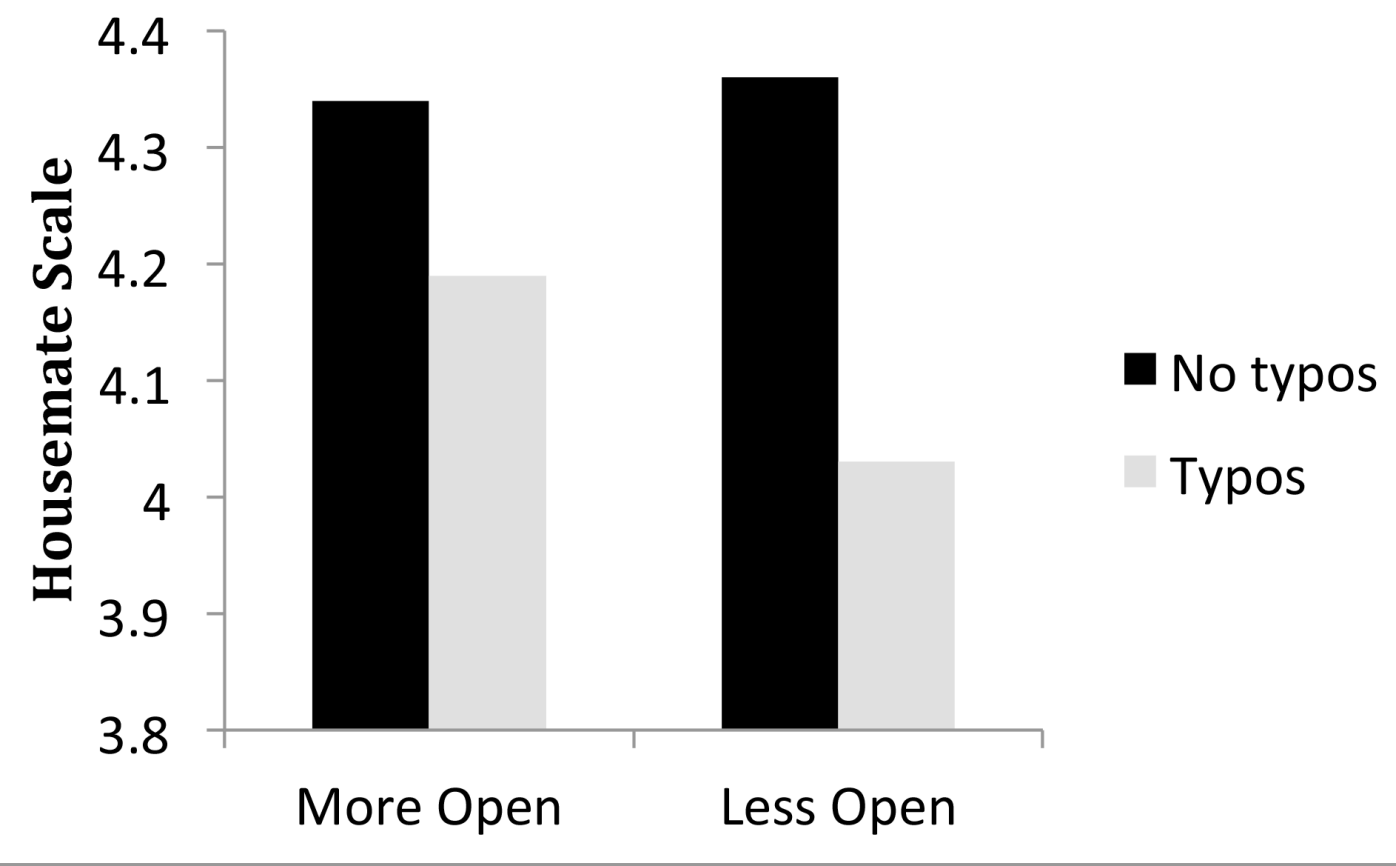
The interaction between Typos and Openness. Participants were divided into two groups, using the Openness median of 3.70. The more open group averaged 4.19 on the Openness scale of the BFI and the less open group averaged 3.15.

**Fig 6:**
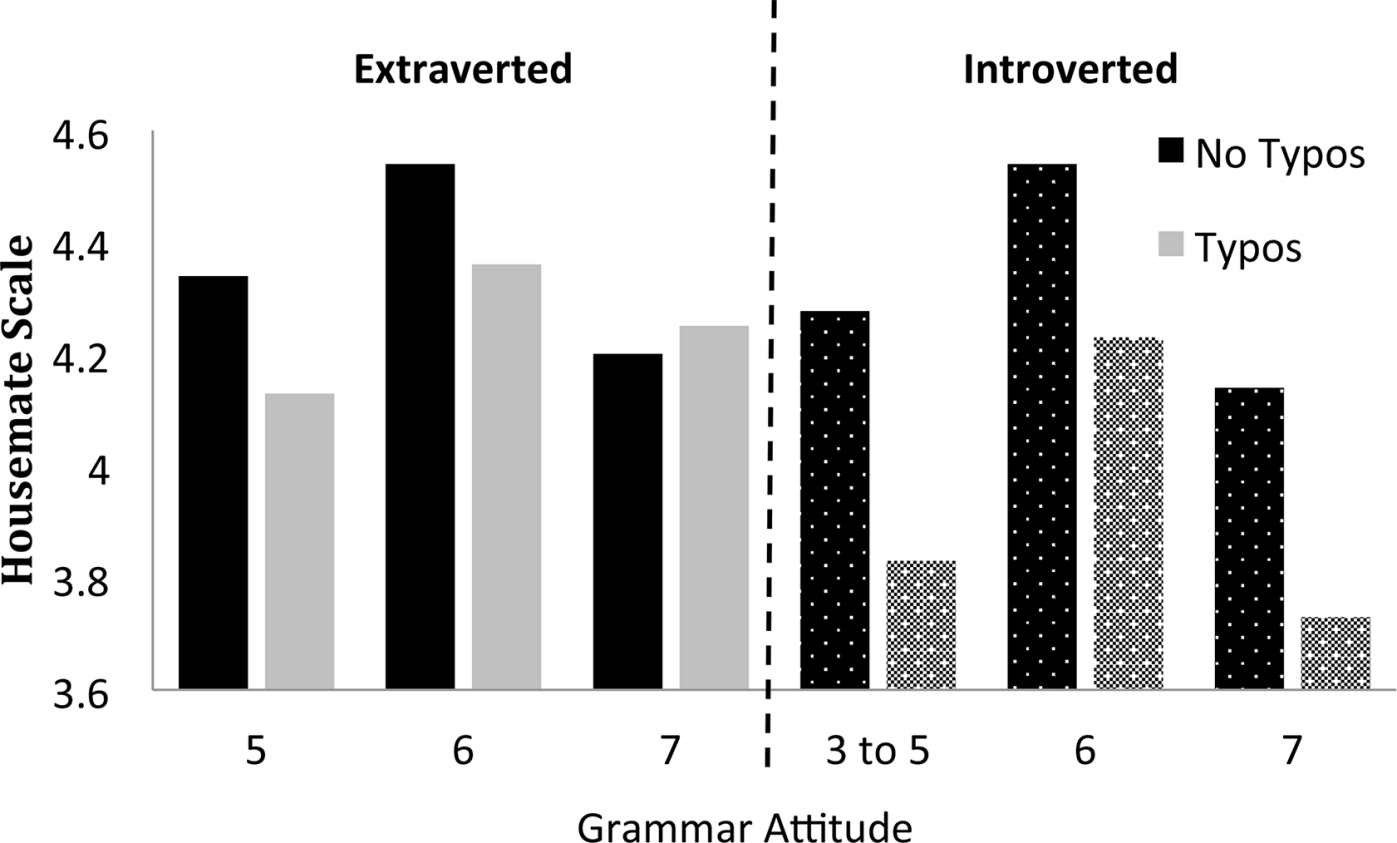
Three-way interaction of Typos, Grammar Attitude, and Extraversion. Introverted participants ratings of grammar attitude ranged from 3 to 7, while extraverts’ scores ranged from 5 to 7.

There was one main effect involving personality and several interactions. In order to illustrate the patterns of interaction for the reader, we have binned the data for Figs 2–6, although the data were not binned in any way for the statistical analysis. We graphed each of the personality interactions that were observed in the statistical model, by splitting participants into two groups (a median split, ignoring participants who scored on the median) for each trait. The figures include items with typos in the “no grammos” condition, and vice versa, in order to more closely reflect the statistical model. If items with the other type of error were excluded from the “no grammos/no typos” bars in the figures, ratings for each of those bars in Figs 2–6 would increase.

Agreeability was the only personality trait to have a main effect on the Housemate Scale. Participants who tested as more agreeable on the BFI tended to rate the paragraphs more positively overall than participants who tested as less agreeable (Fig 2). For each 1 point difference in Agreeability on the BFI, there was a benefit of .30 points (95% CI: .07 to.53 points) on the Housemate Scale.

The overall effect of Agreeability was much larger than any of the interactions with personality, which ranged in magnitude from .03 to .08 of a point on the Housemate Scale. As shown in Fig 2, the participant’s level of Agreeability modulated the impact of Grammos: Less agreeable participants showed more sensitivity to grammos than participants high in agreeability, perhaps because less agreeable people are less tolerant of deviations from convention. In contrast, Agreeability did not modulate the effect of Typos. Extraversion was the only trait that interacted with both types of errors, and the interactions are illustrated in Fig 3. Extroverts were more generous in their assessments of both Grammos and Typos. We had predicted that those higher in Conscientiousness would be more sensitive to the effects of errors in general, but that was not the case. Instead, both highly conscientious participants (Fig 4) and less open participants (Fig 5) had increased sensitivity to Typos, but these personality variables did not impact sensitivity to Grammos. Neuroticism did not impact the Housemate Scale in any measureable way.

Finally, the three-way interaction is illustrated in Fig 6. Surprisingly, extraverts who reported grammar as more important were less sensitive to typos than extraverts who felt good grammar was less important. In contrast, introverts were sensitive to typos across all levels of Grammar Attitude.

Summarizing the results, three out of the Big Five personality traits interacted with only one type of error, either grammos (agreeability) or typos (openness, conscientiousness). One trait (extraversion) interacted with both types of errors, and one trait (neuroticism) interacted with neither. This pattern is consistent with our speculation that typos and grammos carry different evaluative weight and potentially different social meanings.

## Discussion

The primary contribution of the current study is the finding that personality traits influence our reactions to written errors. Participants were told to imagine that the writers of staged email messages were potential housemates; participants evaluated the writers in terms of perceived intelligence, friendliness, and so forth on our Housemate Scale. Ratings were negatively impacted by the presence of either typos or grammos, and ratings were also modulated by personality traits. Although personality traits have been linked to variation in production, particularly the use of specific lexical items [14,16], this is the first study to show that the personality traits of listeners/readers have an effect on the overall assessment—what we might think of as the social processing—of variable language. Different sets of personality traits were relevant for the two types of errors. More extraverted people were likely to overlook written errors that would cause introverted people to judge the person who makes such errors more negatively. Less agreeable people were more sensitive to grammos, while more conscientious and less open people were sensitive to typos.

While there has been a growing body of research examining the social processing of language (e.g., [24–26]), the majority of that work centers on questions related to the variable social positioning of the *speaker*, as opposed to characteristics of the reader/listener, which were our focus here. For instance, Niedzielski shows that people perceive an identical vowel differently when they are given different social information about the presumed speaker [24]. Similarly, Hanulíková et. al. demonstrate that the identification of a speaker as native or non-native has an effect on the neural processing of variable syntactic phenomena as constituting an error [26]. Although the current study does not address language processing directly, our results indicate that variable social and individual properties of listeners/readers may have an effect on how language is processed and assessed.

Much of the research on language comprehension relies on written stimuli because variation in local processing difficulty can easily be measured as fixation time (or reading time) and prosodic variability can be avoided [27–28]. Therefore, it is important to understand whether variation and/or errors in writing result in differential processing costs. And further, are any such costs higher or lower for individuals who differ in a variety of ways, including on personality? If there are processing costs associated with written errors, then our findings make predictions about how those costs might be distributed. For instance, we could predict that participants higher on the agreeableness scale will show a lower processing cost (because they are generally less bothered by written errors of any kind). In general, then, our newfound understanding of the place of personality in the social processing of language points to the need for enhanced models of language production and perception; for more attention to the ways individual variability that can affect language processing; and for more attention to modeling the variation language users encounter, including in written forms of language.

The relationship between personality traits and assessments of written errors thus provides a critical new lens for assessing the place of individual variation in matters of language processing. For instance, given that personality has now been shown to have an effect on both production and comprehension of language, psycholinguists, sociolinguists, and social psychologists can begin to develop hypotheses about how personality affects the mental processes in existing language processing theories. We suspect that the implications will play out differently in constraint-based theories of sentence comprehension, which simultaneously integrate linguistic and nonlinguistic constraints [25,29,30], compared with theories in which language processing is more encapsulated from other types knowledge [31–33]. Thus, the current study points to broader questions about the extent to which language processing is encapsulated and domain-specific.

It remains an open question whether the kind of variation in personality that our participants exhibited affects the most basic aspects of language comprehension (e.g., word recognition, syntactic parsing) or only relatively superficial aspects of interpretation. In psycholinguistic research, this distinction is often characterized as the difference between affecting language processing at the earliest stages, when the utterance is first being perceived, versus affecting more conscious and reflective aspects of processing that are not closely time-locked to the input (e.g., [34–40]). Considering the current results in the context of the existing literature [27–40], it seems very likely that the personality of the reader/listener can impact aspects of pragmatic processing that are not closely time-locked to the input. However, given the role that pragmatic processing can play in lexical and syntactic ambiguity resolution [25,26,29], it is possible that personality could impact language comprehension in a manner that is tightly time-locked to the input as well.

In contrast to the personality variables, demographic, attitude, and behavior variables had very little impact on the Housemate Scale, despite recruiting for a broad range of ages and educational backgrounds in our sample. Notably, we found no impact of time spent on EMC or pleasure reading. Prior research from our lab similarly examined reactions to typos and grammos, and found that participants who engaged more frequently with electronic forms of communication gave higher ratings [6]. The absence of such an effect in the current study may be due to its broader sample of participants. In the current study, the mean EMC score was numerically lower (3.6 vs. 4.2) and the standard deviation was numerically higher (1.59 vs. 0.99) compared with the earlier sample. In the earlier research, all of the participants were college students; within the college population, variability in the use of social media may have clearer implications than in the broader population. For example, investigations of Facebook usage among undergraduates have found that heavy Facebook users tend to be more extraverted [41] and narcissistic [42], and have also found more nuanced relationships between personality and type of Facebook usage [43–46].

In the current study, we observed a three-way interaction involving grammar attitude, but the pattern of interaction was unexpected and difficult to interpret. In that interaction, extraverts who had higher grammar attitude ratings gave paragraphs with typos higher ratings than did extraverts with lower grammar attitude scores. Recall, that extraversion was the only personality trait that interacted with both typos and grammos; however, only the ratings of paragraphs with typos were affected by extraverts’ grammar attitudes. The effect, however, went in the opposite direction of our prediction, which was that grammar attitude would have a negative effect on ratings in general. While it is difficult to know for sure how to interpret this three-way interaction, it could be seen as evidence that extraverts at least interpret typos as more mechanical than grammatical errors, and that those who believe “good grammar is important” don’t view typos as evidence of “bad grammar” and thus do not rate people who produce typos as negatively as extraverts who are less concerned with “good grammar.”

One analytic point that the three-way interaction certainly supports is the contention that typos and grammos are evaluated differently from one another, something we asserted based on the differential relationships between different personality traits and the ratings on the housemate scale. Recall that people with lower agreeability ratings assessed paragraphs with grammos more harshly while more conscientious and less open people assessed paragraphs with more typos more harshly. Similarly, although extraverts were sensitive to both typos and grammos, those extraverts with stronger beliefs about good grammar were more sensitive to typos; however, there were no differences linked to beliefs about good grammar associated with grammos. Thus, we see a further distinction between how typos and grammos are treated.

The effect of typos appears to be more than double the effect of grammos in Fig 1 (est. -.16 vs -.07), counter to our assertion that—when they are noticed—grammos carry more personalized negative attributions than typos. We suspect that the larger effect size for typos reflects the more frequent detection of typos by the reader, a type of processing fluency effect [47–48]. Typos comprised unusual letter strings, while grammos were familiar letter strings, and our earlier research found that the typos were more salient to readers than our grammos [6].

The overall impact of written errors on seemingly unrelated variables such as trustworthiness and consumer purchasing could be related to processing fluency (i.e., readability). Considerable research has manipulated linguistic processing fluency in both spoken and written modalities (e.g., foreign vs. native accents, signal/noise ratio in speech, font used for text) and found surprisingly far-reaching effects [26,47–50]. For example, Song and Schwarz found that a recipe was judged to be tastier and easier to make when presented in a simple font compared with a complex font [48]. Indeed, we suspect that peever errors are annoying, in part, because they disrupt reading fluency. However, processing fluency cannot explain the individual differences summarized above: the personality traits of the reader/listener, quite apart from the processing fluency of the message, impact how grammos and typos impact assessments of writers.

Although the effect sizes in our study were relatively small—typos trials were rated only .47 points lower, overall, on the Housemate Scale and grammos were rated only .22 points lower, the effect size is likely modulated by the density of errors, with bigger effects for larger error/word ratios [20]. Furthermore, small effects still have real-world consequences. For example, Hucks found that typos negatively impacted fulfillment of real-world loan requests [50], while Ghose and Ipeirotis found real-world impacts of written errors on consumer behavior [9]. What is new in the current results is our finding that the personality traits of the reader influence the impact of typos and grammos. As we discuss above, these findings have implications for theories accounting for individual variation in language processing. They also add to the growing literature on the relationship between personality and language [11–16], which until now, has examined only certain aspects of language production, without considering any aspects of language interpretation.

## Supporting Information

S1 Appendix Email Stimuli. (DOCX)

S2 Appendix IRB Waiver. (PDF)

S3 Appendix Dataset. (CSV)

S1 Table Correlations among predictor variables. (DOCX)

S2 Table Descriptive statistics for email questionnaire items. (DOCX)

S3 Table Model 1 Description. (DOCX)
