# Supplementary material for: If You’re House Is Still Available, Send Me an Email: Personality Influences Reactions to Written Errors in Email Messages
Source: PLoS One. 2016 Mar 9;11(3):e0149885. doi: 10.1371/journal.pone.0149885 (PMC4784893; doi:10.1371/journal.pone.0149885)
Supplement: S1 Appendix — (DOCX) [file pone.0149885.s001.docx]

The email stimuli are provided below. There were three versions of each email. One version contained 2-4 grammos (underlined), one version contained 2-4 typos (in boldface), and one version was fully correct (in parentheses).

Hey! My name is Pat and I’m interested in sharing a house with other students who are serious **abuot (about)** there (their) schoolwork but who also know how to relax and have fun.  I like to play tennis and love old school rap.  If your (you’re) someone who likes that kind of thing too, maybe we would **mkae (make)** good housemates.

My name is Chris and I’m **lookign (looking)** for housemates.  It sounds like you are, too.  I’m a junior and am involved in many different activities.  Its (It’s) hard to keep them all straight sometimes and I wonder if I might have been better off to focus more on one or two. Then, my school work wouldn’t of (wouldn’t’ve) suffered like it did last year.  But no big deal, I’ve pulled my GPA up and gotten my professors to help me out.  Their (They’re) really **hepling (helping)** me to do better this term.

Hello, my name is Cameron and I’m a first year **stuednt (student)** in Organizational Studies.  I’m really neat and quiet.  My friends tell me I should of (should’ve) majored in Psychology because I’m **aslo (also)** a really great listener.  I really like to have a clean house and kitchen.  I like music a lot, but I don’t really like for **teh (the)** stereo to be super loud.  If you choose me to be your’re (your) housemate, you won’t regret it.

Hi, I'm Adrian, and I'm looking for some housemates. I love to be in the great outdoors, so if your (you’re) an adventurer, its (it’s) time for us to go **epxlore (explore)**. When I'm not **surroundign (surrounding)** myself with nature, I like to play frisbee or go **runnign (running)**; both are great ways to exercise. And don't worry, I'll wipe the mud off my boots before I go inside!

Hi!  My name is Jamie and I’m ready to move out of the dorms and into a house.  I like cooking, **dacning (dancing)**, playing video games and playing my electric guitar.  I’m in the B-school and taking 18 hours this term.  **Teh (the)** courses are great but their (they’re) also taking a lot of my time.  I could of (could’ve) taken fewer courses but my parents really want me to finish in four years.  If you’re (your) house is still available, send me an e-mail and we can set up a meeting.

Hey, my name is Jessie and I’m an athlete on the UM Swim Team.  I’m also **comited (commited)** to majoring in Kineseology.  Its (It’s) a much tougher major than people think.  Because of my swimming, I’m an early riser, which means I have to go to bed pretty early.  My current housemates don’t like that because it means they have to be quiet.  Their (they’re) always trying to get me to stay up later than I should. Send me an e-mail, **especialyl (especially)** if your (you’re) looking for a great cook who loves to share what they make.

Hey, its (it’s) Alex! I'm always looking to meet new **poeple (people)**, and your (you’re) new, so let's meet. Choosing to live with me will be one of the easiest decisions you'll **mkae (make)** for a long time. I'm looking forward to **haering (hearing)** if your (you’re) going to make the right choice!

My name is Jordan and I'm a sophomore in the Art school. I've got plenty of my old **stfuf (stuff)** that we can hang on our walls, if your (you're) interested. Other than the art, their (there) are a few more hobbies that I'd really like to hang on to. None of them are going to ruin the house, so I can at least guarantee that I'll be a **sfae (safe)** roommate. If you like you're (your) house to feel like a home, I can bring **taht (that)** coziness. Let me know.

My name is C.J. and its (it’s) going to be great to be out of the dorms. I like to practice my **giutar (guitar)**, and their (they’re) less than happy about **teh (the)** loud music where I lived. If your (you’re) into music, and wouldn't mind jamming, hit me back. Trust me, its (it’s) going to be a great year!

Hi there! My name is Sam, and I'm sure you'll enjoy having me as you’re (your) roommate.  I like to cook, and don't mind **doign (doing)** so for vegetarians. Their (they’re) hungry people to (too)! If your (you’re) looking for an easy-going friend who likes to be in the **kichten (kitchen)**, don't lose out on this opportunity.

I’m Taylor and I could be your’re (your) next housemate. I’ve been **livign (living)** in the dorms but things there are getting out of control with all the partying. Students in the dorms aren’t **alwyas (always)** as serious as they need to be about studying **adn (and)** their (they’re) also to (too) loud most of the time.  It’s (its) been a real struggle to try and keep my grades up while living there.  I’m a sophomore with a double **concentartion (concentration)** in Math and Biology, looking for considerate students to live with.

Blair here, and I want to help you pay you’re (your) rent. I’m pretty quiet until the studying is all done. Then its (it’s) time to cut loose! I have a few solid **freinds (friends)**, and their (they’re) usually willing to throw down on the party. Let me **knw (know)** how all this sounds!
