# Supplementary material for: If You’re House Is Still Available, Send Me an Email: Personality Influences Reactions to Written Errors in Email Messages
Source: PLoS One. 2016 Mar 9;11(3):e0149885. doi: 10.1371/journal.pone.0149885 (PMC4784893; doi:10.1371/journal.pone.0149885)
Supplement: S2 Appendix — (PDF) [file pone.0149885.s002.pdf]

UNIVERSITY OF MICHIGAN
eResearch.umich.edu

Health Sciences and Behavioral Sciences Institutional Review Board • 540 East Liberty Street, Suite 202, Ann Arbor, MI 48104-2210 • phone (734) 936-0933 • fax (734) 998-9171 • irbhsbs@umich.edu

---

To: Julie Boland

From:

Richard Redman

Cc:

Julie Boland

Subject: Notice of Exemption for [HUM00062035]

SUBMISSION INFORMATION:
Title: Gathering Linguistic Judgments on Mechanical Turk
Full Study Title (if applicable): Gathering Linguistic Judgments on Mechanical Turk
Study eResearch ID: HUM00062035
Date of this Notification from IRB: 3/12/2012
Date of IRB Exempt Determination: 3/12/2012
UM Federalwide Assurance: FWA00004969 expiring on 6/13/2014
OHRP IRB Registration Number(s): IRB00000246

IRB EXEMPTION STATUS:
The IRB HSBS has reviewed the study referenced above and determined that, as currently described, it is exempt from ongoing IRB review, per the following federal exemption category:

> **EXEMPTION #2 of the 45 CFR 46.101.(b):**
> Research involving the use of educational tests (cognitive, diagnostic, aptitude, achievement), survey procedures, interview procedures or observation of public behavior, unless: (i) information obtained is recorded in such a manner that human subjects can be identified, directly or through identifiers linked to the subjects; and (ii) any disclosure of the human subjects' responses outside the research could reasonably place the subjects at risk of criminal or civil liability or be damaging to the subjects' financial standing, employability, or reputation.

Note that the study is considered exempt as long as any changes to the use of human subjects (including their data) remain within the scope of the exemption category above. Any proposed changes that may exceed the scope of this category, or the approval conditions of any other non-IRB reviewing committees, must be submitted as an amendment through eResearch.

Although an exemption determination eliminates the need for ongoing IRB review and approval, you still have an obligation to understand and abide by generally accepted principles of responsible and ethical conduct of research.

Examples of these principles can be found in the Belmont Report as well as in guidance from professional societies and scientific organizations.

SUBMITTING AMENDMENTS VIA eRESEARCH:
You can access the online forms for amendments in the eResearch workspace for this exempt study, referenced above.

ACCESSING EXEMPT STUDIES IN eRESEARCH:
Click the "Exempt and Not Regulated" tab in your eResearch home workspace to access this exempt study.

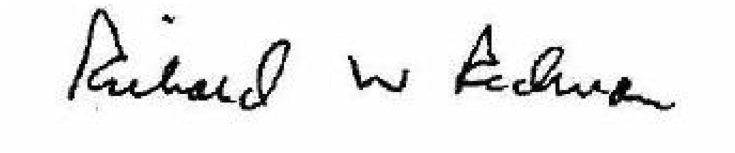

Richard Redman
Chair, IRB HSBS
