# Supplementary material for: If You’re House Is Still Available, Send Me an Email: Personality Influences Reactions to Written Errors in Email Messages
Source: PLoS One. 2016 Mar 9;11(3):e0149885. doi: 10.1371/journal.pone.0149885 (PMC4784893; doi:10.1371/journal.pone.0149885)
Supplement: S1 Table — (DOCX) [file pone.0149885.s004.docx]

**S1 Table. Correlation Coefficients (r) between Personality Variables and Demographic/Behavioral Variables.**

| **Trait** | **Education** | **Age** | **Reads** | **E-comm** | **Gr.Att** | **Bothered** |
| --- | --- | --- | --- | --- | --- | --- |
| Extraversion | .03 | .11 | .12 | .33 | .25 | -.14 |
| Agreeable | .10 | .24 | -.03 | .00 | .26 | -.24 |
| Conscientious | .21 | .26 | .08 | .02 | .33 | .07 |
| Neurotic | -.12 | -.31 | .04 | -.10 | -.14 | .03 |
| Open | -.06 | -.01 | .25 | .11 | .14 | .13 |
