# Supplementary material for: If You’re House Is Still Available, Send Me an Email: Personality Influences Reactions to Written Errors in Email Messages
Source: PLoS One. 2016 Mar 9;11(3):e0149885. doi: 10.1371/journal.pone.0149885 (PMC4784893; doi:10.1371/journal.pone.0149885)
Supplement: S2 Table — (DOCX) [file pone.0149885.s005.docx]

**Table S2. Summary Statistics for the 10 items comprising the Housemate Scale.**

| **Question** | **Mean** | **Standard deviation** | **Skewness** | **Kurtosis** |
| --- | --- | --- | --- | --- |
| **Friends with** | 4.19 | 1.47 | -0.28 | -0.64 |
| **Housemate** | 4.28 | 1.54 | -0.27 | -0.67 |
| **Similar** | 3.50 | 1.59 | 0.21 | -0.90 |
| **Friendly** | 5.15 | 1.09 | -0.97 | 1.85 |
| **Sophisticated** | 3.34 | 1.26 | 0.19 | -0.20 |
| **Intelligent** | 4.19 | 1.39 | -0.08 | -0.56 |
| **Conscientious** | 4.33 | 1.49 | -0.33 | -0.58 |
| **Considerate** | 4.65 | 1.43 | -0.48 | -0.39 |
| **Likeable** | 4.80 | 1.23 | -0.72 | 0.61 |
| **Trustworthy** | 4.50 | 1.26 | -0.35 | 0.26 |
