# Supplementary material for: If You’re House Is Still Available, Send Me an Email: Personality Influences Reactions to Written Errors in Email Messages
Source: PLoS One. 2016 Mar 9;11(3):e0149885. doi: 10.1371/journal.pone.0149885 (PMC4784893; doi:10.1371/journal.pone.0149885)
Supplement: S3 Table — (DOCX) [file pone.0149885.s006.docx]

**Table S3. Summary of Model 1, without Personality Variables.**

| Effect | Estimate | 95% Confidence Interval | | F |
| --- | --- | --- | --- | --- |
| **Typos** | **-.13** | **-.17** | **-.08** | **19.07** |
| **Grammos** | **-.07** | **-.11** | **-.04** | **15.79** |
| Age | -.01 | -.03 | .00 | 1.59 |
| Education | .07 | -.05 | .19 | 0.01 |
| EMC | .06 | -.03 | .15 | 0.51 |
| Pleasure Reading | -.03 | -.11 | .06 | 0.54 |
| Grammar Attitude | -.05 | -.21 | .11 | 0.03 |
| **Typos X Grammar Attitude** | **.06** | **.01** | **.11** | **6.19** |
| Grammos X Education | -.03 | -.06 | .00 | 3.29^M^ |

*Note:* Significant effects and interactions are bolded. Marginally significant effects are labelled with a superscripted M. Interactions that did not approach significance are not included in the table.
